# Protective role of cellular prion protein against TNFα-mediated inflammation through TACE α-secretase

**DOI:** 10.1038/s41598-017-08110-x

**Published:** 2017-08-09

**Authors:** Juliette Ezpeleta, François Boudet-Devaud, Mathéa Pietri, Anne Baudry, Vincent Baudouin, Aurélie Alleaume-Butaux, Nathalie Dagoneau, Odile Kellermann, Jean-Marie Launay, Benoit Schneider

**Affiliations:** 1 INSERM, UMR-S 1124, F-75006 Paris, France; 2 Université Paris Descartes, Sorbonne Paris Cité, UMR-S 1124, F-75006 Paris, France; 3 AP-HP, INSERM UMR-S 942, Hôpital Lariboisière, F-75010 Paris, France; 4 Pharma Research Department, Hoffmann-La-Roche Ltd, CH4070 Basel, Switzerland

## Abstract

Although cellular prion protein PrP^C^ is well known for its implication in Transmissible Spongiform Encephalopathies, its functions remain elusive. Combining *in vitro* and *in vivo* approaches, we here show that PrP^C^ displays the intrinsic capacity to protect neuronal cells from a pro-inflammatory TNFα noxious insult. Mechanistically, PrP^C^ coupling to the NADPH oxidase-TACE α-secretase signaling pathway promotes TACE-mediated cleavage of transmembrane TNFα receptors (TNFRs) and the release of soluble TNFR, which limits the sensitivity of recipient cells to TNFα. We further show that PrP^C^ expression is necessary for TACE α-secretase to stay at the plasma membrane in an active state for TNFR shedding. Such PrP^C^ control of TACE localization depends on PrP^C^ modulation of β1 integrin signaling and downstream activation of ROCK-I and PDK1 kinases. Loss of PrP^C^ provokes TACE internalization, which in turn cancels TACE-mediated cleavage of TNFR and renders PrP^C^-depleted neuronal cells as well as PrP^C^ knockout mice highly vulnerable to pro-inflammatory TNFα insult. Our work provides the prime evidence that in an inflammatory context PrP^C^ adjusts the response of neuronal cells targeted by TNFα through TACE α-secretase. Our data also support the view that abnormal TACE trafficking and activity in prion diseases originate from a-loss-of-PrP^C^ cytoprotective function.

## Introduction

Chronic neuroinflammation is a hallmark of several neurodegenerative disorders such as Alzheimer’s or Parkinson’s diseases that relies on the long-standing activation of microglia and astrocytes in the central nervous system (CNS). These cells produce neurotoxic mediators, such as pro-inflammatory cytokines (TNFα) and interleukins (IL1, IL6) that contribute to dysfunction and degeneration of diseased neurons. The release of pro-inflammatory mediators by microglia also favors the permeabilisation of the blood brain barrier and the subsequent infiltration of peripheral leukocytes, including T cells and macrophages that amplify the disease states (for review see ref. 1).

The cellular prion protein PrP^C^, which is mainly known for its role in Transmissible Spongiform Encephalopathies (TSEs), was shown to exert protective effect against inflammation. Indeed, PrP^C^ displays the intrinsic capacity to modulate in mice the lipopolysaccharide-induced activation of both microglia in the CNS and macrophages in the periphery^2^. In immune cells, PrP^C^ was also reported to balance the release of pro-inflammatory factors during the acute phase of bacterial infection with the production of anti-inflammatory cytokines during the later stage of infection^3^. However, it remains unknown whether PrP^C^ protection against inflammation would also depend on PrP^C^ capacity to adjust the response of cells targeted by pro-inflammatory factors.

PrP^C^ is a ubiquitous protein that is more abundantly expressed in neurons. It is a GlycosylPhosphatidylInositol(GPI)-anchored protein tethered to the outer leaflet of the plasma membrane. The presence of PrP^C^ in detergent-resistant microdomains, *i.e*. lipid-rafts or caveolae, of the plasma membrane, and interaction with several partners fits in with the notion that PrP^C^ is physiologically associated with signaling events and behaves as receptor or co-receptor or as a scaffolding protein regulating the assembly of diverse interactors and signaling modules^4, 5^. This includes β1 integrins as loss of PrP^C^ in neural stem cells leads to micro-aggregation of β1 integrins at the plasma membrane and a rise of β1 integrin signaling that promotes overactivation of the rhoA-associated coiled-coil containing kinase (ROCK) and impairs neuritogenesis^6^. Besides, using an antibody-based approach to mimic an activation signal for PrP^C^, we provided evidence that PrP^C^ can activate in neuronal cells several signaling effectors such as the Fyn tyrosine kinase^7^ and NADPH oxidase^8^. Reactive oxygen species (ROS) produced by NADPH oxidase are not toxic and act as second message signals involved in the activation of TACE α-secretase, a member of a disintegrin and metalloproteinase (ADAM) family^9, 10^. Via its coupling to NADPH oxidase, PrP^C^ stimulates TACE-dependent cleavage of transmembrane pro-TNFα into soluble TNFα (sTNFα) that behaves in neuronal cells as an autocrine modulator of neurotransmitter-associated functions through binding to plasma membrane TNFα receptors (TNFRs)^9^. Of note, TACE was shown to also assume the cleavage of TNFRs in fibroblasts, leukocytes or neuronal cells^11–13^. This raises the possibility that PrP^C^ would exert protection against the pro-inflammatory TNFα cytokine by balancing the TACE-dependent release of sTNFα with the TACE-dependent shedding of TNFRs.

In this study, we show that PrP^C^ positively contributes to the shedding of plasma membrane TNFRs through its coupling to the NADPH oxidase-TACE α-secretase pathway in neuronal stem cells, their neuronal derivatives and primary cerebellar granule neurons. The cytoprotective effect of PrP^C^ against sTNFα also depends on a PrP^C^ control of TACE localization at the plasma membrane. Cell depletion of PrP^C^ (PrP^null^-cells) provokes the internalization of TACE, which diverts TACE activity away from TNFR substrate that accumulates at the plasma membrane and renders PrP^null^-cells highly sensitive to exogenous sTNFα. We show that TACE internalization in PrP^null^-cells relates to a loss of PrP^C^ regulatory function towards plasma membrane β1 integrins and downstream signaling. Finally, we substantiate in PrP^C^ knockout mice that such deregulation of the TACE-TNFR pathway in the brain is at the root of exaggerated sensitivity to sTNFα noxious insult. Our work thus unravels a new role for PrP^C^ signaling related to cytoprotection against sTNFα-mediated inflammation.

## Results

### PrP^C^ coupling to the NADPH oxidase-TACE signaling pathway promotes TNFR1 shedding

Because the cell sensitivity to sTNFα depends on the amount of TNFRs present at the plasma membrane, we first sought to determine whether PrP^C^ would impact on cell surface TNFR level focusing on TNFR type 1 (TNFR1), a transmembrane trimeric receptor composed of three identical subunits, that mainly relays sTNFα toxicity^14^.

**Figure 1:**
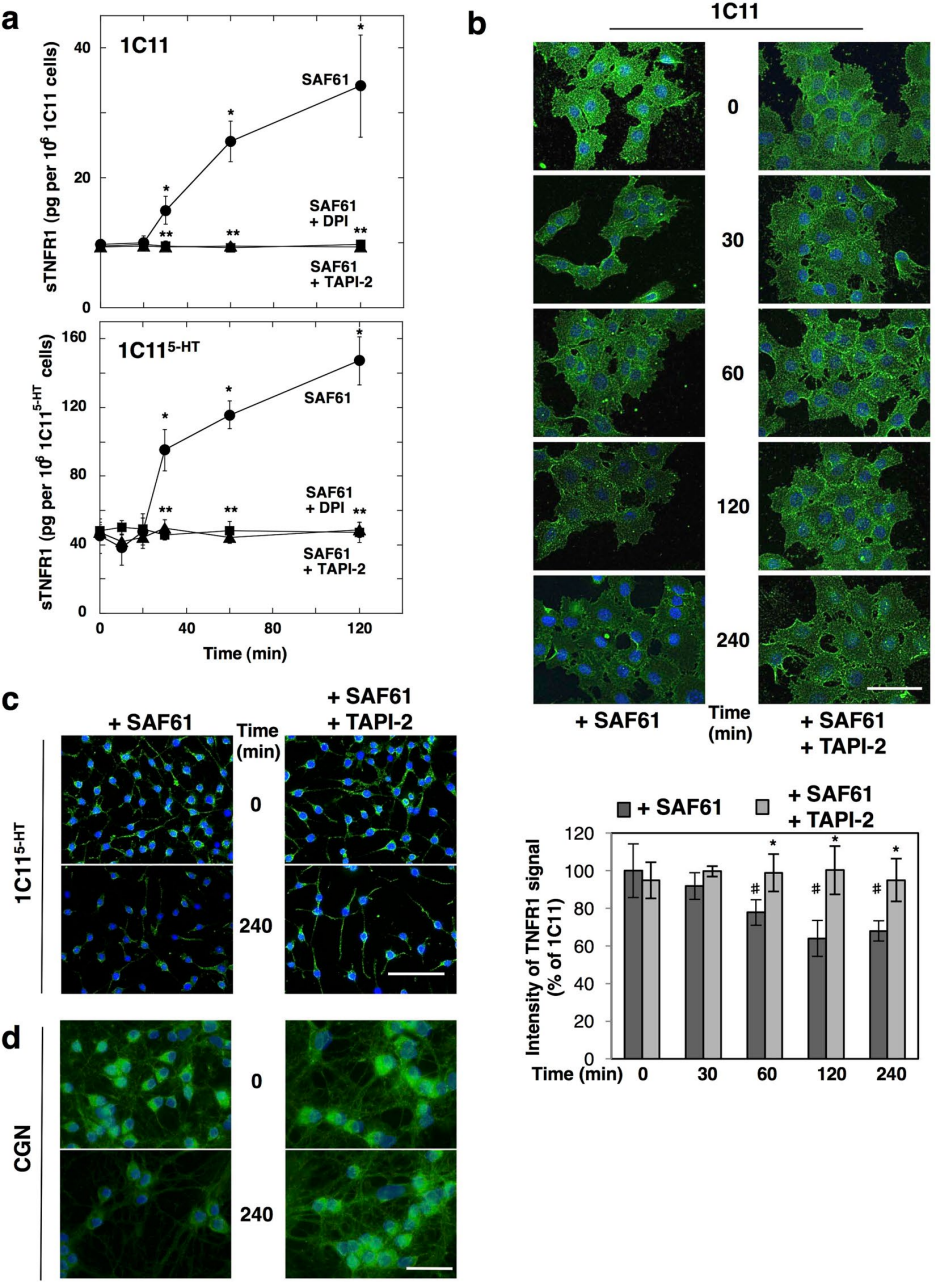
PrP^C^ coupling to the NADPH oxidase-TACE signaling pathway promotes TNFR1 shedding. (**a**) Time-course accumulation of sTNFR1 in the cell culture medium of 1C11 cells and 1C11^5-HT^ neuronal cells upon PrP^C^ ligation with SAF61 PrP antibody (10 μg ml^−1^). TNFR1 shedding induced by PrP antibodies is abolished upon inhibition of NADPH oxidase with DPI (100 μM) or TACE with TAPI-2 (100 μM). *p < 0.01 *vs*. nontreated cells. **p < 0.01 *vs*. cells exposed to SAF61 antibody. (**b**–**d**) Immunofluorescence experiments and quantification histograms showing progressive TNFR1 depletion at the cell surface of 1C11 cells (**b**), 1C11^5-HT^ cells (**c**), and primary CGNs (**d)** exposed to SAF61 PrP antibody and cancellation upon addition of TAPI-2. Scale bar = 50 μm. ^#^ *p* < 0.05 *vs*. nontreated cells. *p < 0.05 *vs*. cells exposed to SAF61 antibody alone. Data shown are the mean ± SEM from three experiments performed in triplicate.

Antibody mediated-PrP^C^ ligation, used to mimic an activation signal for PrP^C^ ^5, 7^, elicited the release of soluble TNFR1 (sTNFR1) into the culture media of 1C11 neuroectodermal cells and their serotonergic 1C11^5-HT^ neuronal derivatives^15^ as assessed by ELISA (Fig. 1a). sTNFR1 was detected as soon as 30 min after PrP^C^ ligation with SAF61 antibody (10 µg ml^−1^). ELISA-based quantification of monomeric TNFR1 subunit in the culture medium revealed that the level of sTNFR1 reached at 120 min was ~4-fold above basal level (Fig. 1a). Concomitantly, immunofluorescence experiments that detect the trimeric form of TNFR1 at the plasma membrane showed that PrP^C^ ligation triggered a time-dependent depletion of TNFR1 at the cell surface with a maximum immunostaining decrease (~1.5-fold) reached by 120 min exposure to SAF61 antibody (Fig. 1b,c). Inhibition of NADPH oxidase (diphenyleneiodonium- DPI, 100 µM) or TACE (TAPI-2, 100 µM) abrogated the shedding of TNFR1 induced by PrP^C^ ligation (Fig. 1a–c), indicating that PrP^C^ coupling to the NADPH oxidase-TACE α-secretase signaling pathway controls cell surface TNFR1 level in 1C11 neuronal stem cells and their serotonergic progenies. Beyond the direct activation of TACE α-secretase by ROS through modification of the valence state of Zn^2+^ at the catalytic site^16^, redox activation of guanylate cyclase (GC) and subsequent production of cGMP was also shown to promote TACE-dependent TNFR1 shedding in sepsis conditions^17^. We thus probed the involvement of GC in PrP^C^-induced TNFR1 shedding by inhibiting GC with NS-2028 (1 µM). We found that GC inhibition had no impact on sTNFR1 release induced by SAF61 antibody in 1C11 precursor cells (Supplementary Fig. 1a), while TNFR1 shedding was 40% decreased in 1C11^5-HT^ cells (Supplementary Fig. 1b). This result suggests that guanylate cyclase is a potential signaling intermediate in PrP^C^ coupling to TACE α-secretase in 1C11^5-HT^ neuronal cells only.

ADAM10 and γ-secretase have also been involved in the shedding of TNFR1^18, 19^. We thus probed the implication of these two proteases in the PrP^C^-regulated cleavage of TNFR1 using the pharmacological inhibitors GI254023X (50 nM) for ADAM10 and DAPT (10 µM) for γ-secretase. As assessed through the measure of sTNFR1 level in the cell culture medium, the inhibition of ADAM10 or γ-secretase had no effect on the release of TNFR1 ectodomain induced by PrP^C^ ligation with SAF61 antibody in 1C11 and 1C11^5-HT^ cells (Supplementary Fig. 1a,b). This indicates that ADAM10 and γ-secretase play no role in PrP^C^-stimulated TNFR1 shedding.

Finally, we extended our study to other cell systems and found that PrP^C^-dependent control of TACE-mediated TNFR1 shedding also occurred in primary cultures of cerebellar granule neurons (CGNs). Exposure of CGNs to PrP antibodies (SAF61 10 µg ml^−1^) triggered a time-dependent decrease in the level of TNFR1 at the neuronal cell surface up to 240 min (Fig. 1d), which was cancelled by inhibiting TACE activity (Fig. 1d).

As a whole, these data indicate the amount of TNFR1 molecules expressed at the plasma membrane of neuronal stem cells and neurons depends on PrP^C^ signaling that stimulates TNFR1 shedding via the NADPH oxidase-TACE α-secretase cascade.

### PrP^C^-dependent regulation of TNFR1 shedding governs cell sensitivity to sTNFα toxicity

As PrP^C^ intrinsically stimulates TACE-mediated TNFR1 shedding, we next assessed whether PrP^C^ confers cell protection against sTNFα toxicity. To address such PrP^C^ function, we exploited siRNA-mediated PrP^C^ silenced cells and primary neurons from PrP^0/0^ mice and compared the sensitivity to sTNFα of PrP^C^-depleted cells to that of their corresponding PrP^C^ expressing counterparts.

**Figure 2:**
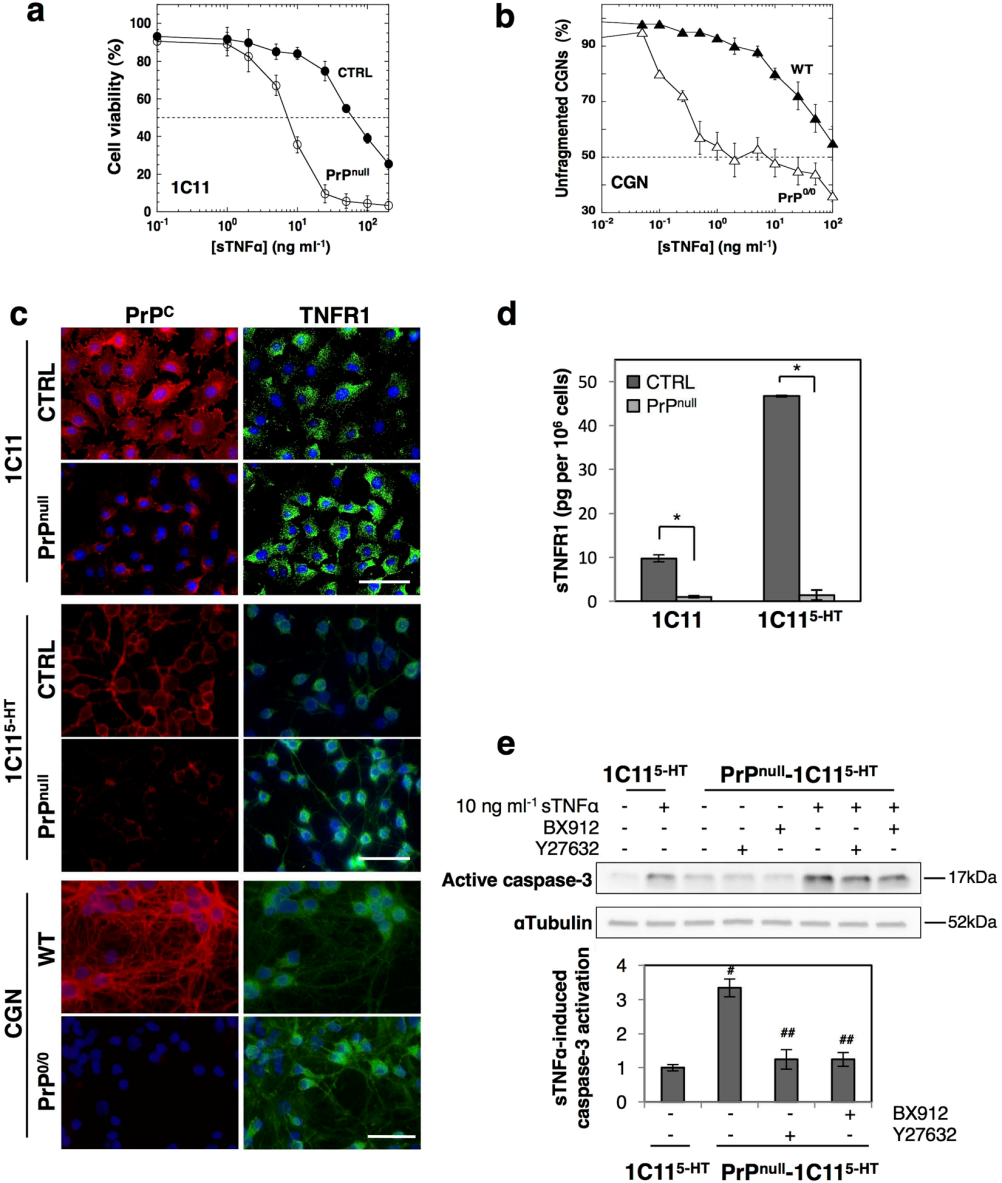
Loss of PrP^C^ exacerbates cell sensitivity to sTNFα by reducing TNFR1 shedding in a ROCK-I- and PDK1-dependent manner. (**a**) Reduced viability of PrP^null^-1C11 cells after exposure to increasing sTNFα concentrations for 72 h as compared to 1C11 cells (CTRL). (**b**) Increased dendritic fragmentation in PrP^0/0^-CGNs after exposure to increasing concentrations of sTNFα for 72 h compared to wild-type CGNs (WT). For figures **a** and **b**, LD_50_^TNFα^ values are indicated in Table 1. (**c**) Immunofluorescence experiments showing enhanced level of TNFR1 at the cell surface of PrP^null^-1C11 and PrP^null^-1C11^5-HT^ cells as well as PrP^0/0^ CGNs as compared to their corresponding PrP^C^ expressing cells (CTRL, WT). Scale bar = 50 µm. **(d)** ELISA-based quantification experiments indicating reduced concentration of sTNFR1 in the culture medium of PrP^null^-1C11/1C11^5-HT^ cells compared to PrP^C^ expressing cells. *p < 0.01. **(e)** Western blots showing a stronger activation of caspase-3 in PrP^null^-1C11^5-HT^ cells exposed to sTNFα (10 ng ml^−1^) for 120 min than in PrP^C^ expressing cells. Antagonizing either ROCK activity with Y-27632 (100 µM for 1 h) or PDK1 activity with BX912 (1 µM for 1 h) in PrP^C^-depleted cells reduces toxic action of sTNFα. ^#^ *p* < 0.05 *vs*. PrP^C^-expressing cells exposed to sTNFα. ^##^ *p* < 0.05 *vs*. PrP^null^-cells treated with sTNFα. Data shown are the mean ± SEM from three experiments performed in triplicate.

We first determined the dose of exogenous sTNFα that induces 50% cell death (LD_50_^TNFα^) of 1C11 precursor cells, serotonergic 1C11^5-HT^ neural cells and their counterparts silenced for PrP^C^ expression (PrP^null^-cells). As shown in Fig. 2a and Table 1, PrP^null^-1C11 and 1C11^5-HT^ cells were ~5- to 9-fold more sensitive to a 48 h exposure to sTNFα than their corresponding PrP^C^ expressing cells. In primary cultures of CGNs, we also found a PrP^C^ role in the control of cell sensitivity to sTNFα. In this set of experiments, we determined the dose of exogenous sTNFα inducing neuronal dysfunction for 50% of CGNs through dendrite fragmentation^13^. We monitored that CGNs isolated from PrP^0/0^-FVB mice were ~20-fold more sensitive to a 48 h exposure to sTNFα than PrP^C^-expressing CGNs (Fig. 2b and Table 1).

**Table 1:** Impact of PrP^C^ depletion on cell sensitivity to sTNFα in 1C11 precursor cells, 1C11^5-HT^ neuronal cells and primary CGNs.

	LD_50_^TNFα^ (ng ml^−1^)	
	WT	PrP^null^/PrP^0/0^
1C11	70 ± 10	8.1 ± 1.5
1C11^5-HT^	8.1 ± 1.2	1.5 ± 0.3
CGN	100 ± 20	5.2 ± 3.1

LD_50_^TNFα^ values correspond to the concentration of sTNFα inducing a 50% cell death in 1C11 and 1C11^5-HT^ cells or inducing dendritic fragmentation for 50% of neuronal cells in CGNs. Data are the mean ± SEM of three independent experiments performed in triplicate.

Through immunofluorescence experiments, we recorded an increase of TNFR1 at the plasma membrane of PrP^null^-1C11 and 1C11^5-HT^ cells as well as PrP^0/0^-CGNs compared to their wild-type counterparts (Fig. 2c). Of note, the level of sTNFR1 in the cell culture medium of PrP^null^-1C11 and 1C11^5-HT^ cells was ∼10- to 25-fold lower than that measured with wild type cells (Fig. 2d), indicating reduced shedding of cell surface TNFR1 in the absence of PrP^C^.

Corroborating the augmentation of plasma membrane TNFR1 level in PrP^null^-1C11/1C11^5-HT^ cells and PrP^0/0^-CGNs associated with the increased vulnerability of PrP^C^-depleted cells to sTNFα, we found that TNFR1 signaling is exacerbated in the absence of PrP^C^. In response to sTNFα exposure for 2 h, the activation of caspase-3, a downstream effector of TNFR1 signaling^20^, was ∼2 to 4-fold enhanced in PrP^null^-1C11 (Supplementary Fig. 2) and PrP^null^-1C11^5-HT^ cells (Fig. 2e) compared to wild type cells.

Loss of PrP^C^ therefore triggers a deficit of TNFR1 shedding, leading to plasma membrane accumulation of TNFR1 and enhanced TNFR1 death signaling, that renders PrP^C^-depleted cells highly vulnerable to sTNFα toxicity. Our data thus argue for a protective function of PrP^C^ against sTNFα-associated inflammation.

### Cancellation of TNFR1 shedding in PrP^null^-cells is associated with TACE internalization induced by ROCK-I and PDK1 kinases

**Figure 3:**
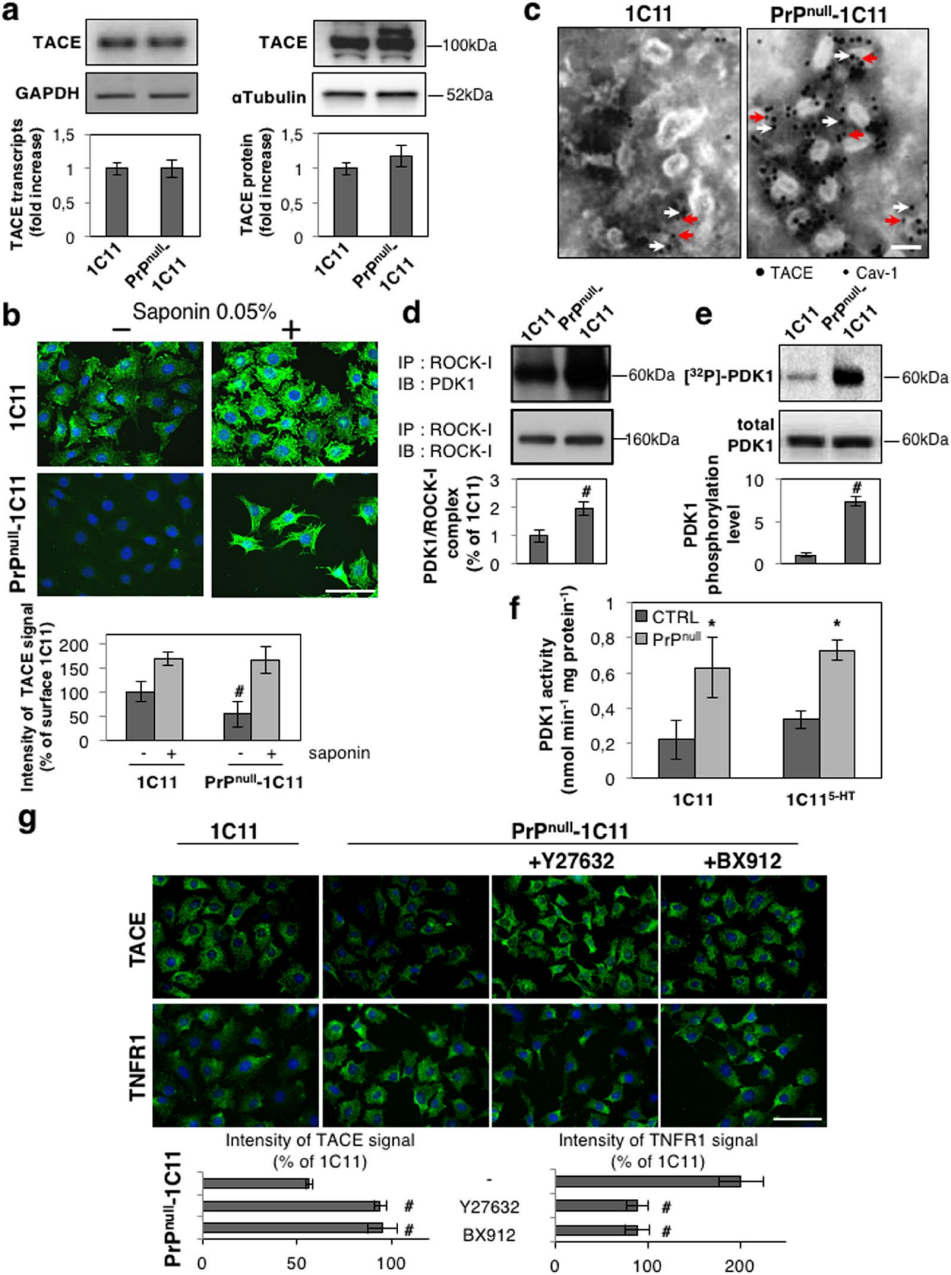
Overactivation of the ROCK-I-PDK1 signaling module in the absence of PrP^C^ promotes TACE internalization. (**a**) RT-PCR (left) and Western-blot (right) experiments showing that siRNA-based PrP^C^ silencing in 1C11 cells does not impact on TACE expression at the mRNA and protein levels. (**b**) Immunolabeling experiments indicating that TACE level is reduced at the cell surface of PrP^null^-1C11 cells *vs*. 1C11 cells. Cell permeabilization with saponin reveals that TACE is internalized in the absence of PrP^C^. Scale bar = 50 µm. ^#^ *p* < 0.05 *vs*. PrP^C^ expressing 1C11 cells. (**c**) Transmission electron micrographs showing TACE (7-nm gold particles, white arrows) accumulation in Caveolin-1-enriched vesicles (labeled by 5-nm gold particles, red arrows) in PrP^null^-1C11 cells. Scale bar = 100 nm. **(d)** Immunoprecipitation of ROCK-I followed by immunoblotting of PDK1 reveals enhanced interaction between ROCK-I and PDK1 in PrP^null^-1C11 cells compared to 1C11 cells. ^#^ *p* < 0.05. (**e**) Cell ^32^P metabolic labeling followed by PDK1 immunoprecipitation and western blotting indicates higher PDK1 phosphorylation level in PrP^null^-1C11 cells than in 1C11 cells. ^#^ *p* < 0.05. (**f**) Augmented PDK1 activity in PrP^null^- *vs*. 1C11/1C11^5-HT^ cells. *p < 0.01 *vs*. PrP^C^ expressing cells. (**g**) Immunolabelings of cell surface TACE and TNFR1 showing that inhibition of either ROCK (Y-27632 100 µM for 2 h) or PDK1 (BX912 1 µM for 2 h) targets TACE back to the plasma membrane of PrP^null^-1C11 cells and rescues TNFR1 shedding. Scale bar = 50 µm. ^#^ *p* < 0.05 *vs*. nontreated cells. Data shown are the mean ± SEM from three experiments performed in triplicate.

Defect in TNFR1 shedding caused by the absence of PrP^C^ prompted us to examine the status of the TACE α-secretase in PrP^null^-cells. While no significant variation in TACE expression was measured at the mRNA and protein levels between PrP^null^- and PrP^C^-expressing cells (Fig. 3a), immunolabeling experiments revealed that TACE was quite absent at the plasma membrane of PrP^null^-cells but found intracellularly after cell permeabilization with saponin 0.05% (Fig. 3b). Transmission electron microscopy experiments further indicated that TACE was internalized in vesicles enriched with the caveolin-1 protein (Cav-1) in PrP^null^-1C11 cells (Fig. 3c). These observations suggest that loss of PrP^C^ promotes TACE internalization.

Such internalization of TACE in PrP^C^-depleted cells is reminiscent of what we observed in prion-infected neurons^13, 21^. We showed that pathogenic prions (PrP^Sc^) overstimulate ROCK-I, which binds and phosphorylates PDK1, leading to PDK1 overactivity^21^. Overstimulated PDK1 promotes the phosphorylation and displacement of TACE from the plasma membrane to intracellular Cav-1-enriched vesicles in prion-infected neurons^13^. We thus probed whether the internalization of TACE and subsequent deficit in TNFR1 shedding in PrP^null^-cells would also relate to overactivation of the ROCK-I/PDK1 duo.

Immunoprecipitation experiments revealed that the pool of PDK1 molecules interacting with ROCK-I subtype was ~2-fold more abundant in PrP^null^-1C11 cells than in PrP^C^ expressing cells (Fig. 3d). Such enhanced interaction between ROCK-I and PDK1 in PrP^null^-1C11 cells was accompanied by an increased PDK1 phosphorylation level (Fig. 3e), leading to a ~2- to 3-fold increase in PDK1 activity in PrP^null^-cells compared to their PrP^C^ expressing counterparts (Fig. 3f).

In PrP^null^-cells, overactivation of the ROCK-I/PDK1 module compromises TACE localization at the plasma membrane. The inhibition of either ROCK-I with Y-27632 (100 µM) or PDK1 with BX912 (1 µM) for 1 h indeed allowed to direct TACE back to the plasma membrane of PrP^null^-cells (Fig. 3g). In addition, inhibition of ROCK-I or PDK1 in PrP^null^-cells rescued TACE cleavage activity towards TNFR1 as assessed by reduced level of TNFR1 at the plasma membrane (Fig. 3g) and desensitization of PrP^null^-1C11 and 1C11^5-HT^ cells from sTNFα-induced caspase-3 activation (Fig. 2e and Supplementary Fig. 2).

These results indicate that in the absence of PrP^C^ overactivated ROCK-I and PDK1 kinases promote the internalization of TACE and neutralize TACE activity towards TNFR1. Beyond PrP^C^ capacity to stimulate TACE-mediated TNFR1 shedding, the protective role of PrP^C^ against sTNFα further depends on PrP^C^ ability to maintain TACE α-secretase at the cell surface in an active state for TNFR1 cleavage.

### The presence of active TACE α-secretase at the plasma membrane depends on PrP^C^-mediated regulation of β1 integrin signaling

In lipid rafts of the plasma membrane, PrP^C^ is assumed to function as a dynamic platform for the assembly and modulation of the signaling activity of various modules^4^. Such PrP^C^ role possibly relies on the interaction between PrP^C^ and the membrane protein Cav-1^7, 22^ that also mediates the recruitment of β1 integrins to rafts and activates β1 integrin signaling^23, 24^. By controlling Cav-1 availability for β1 integrins^22^, PrP^C^ exerts a negative regulatory action on β1 integrin signaling^6^. Such interplay between PrP^C^ and β1 integrins in 1C11 neuronal stem cells and PC12 cells fine-tunes the ROCK activity necessary for neurite sprouting^6, 25^. We next wondered whether loss of PrP^C^ modulatory action on β1 integrin signaling would account for the ROCK-I/PDK1-dependent TACE internalization and subsequent defect in TNFR1 shedding in PrP^null^-cells.

**Figure 4:**
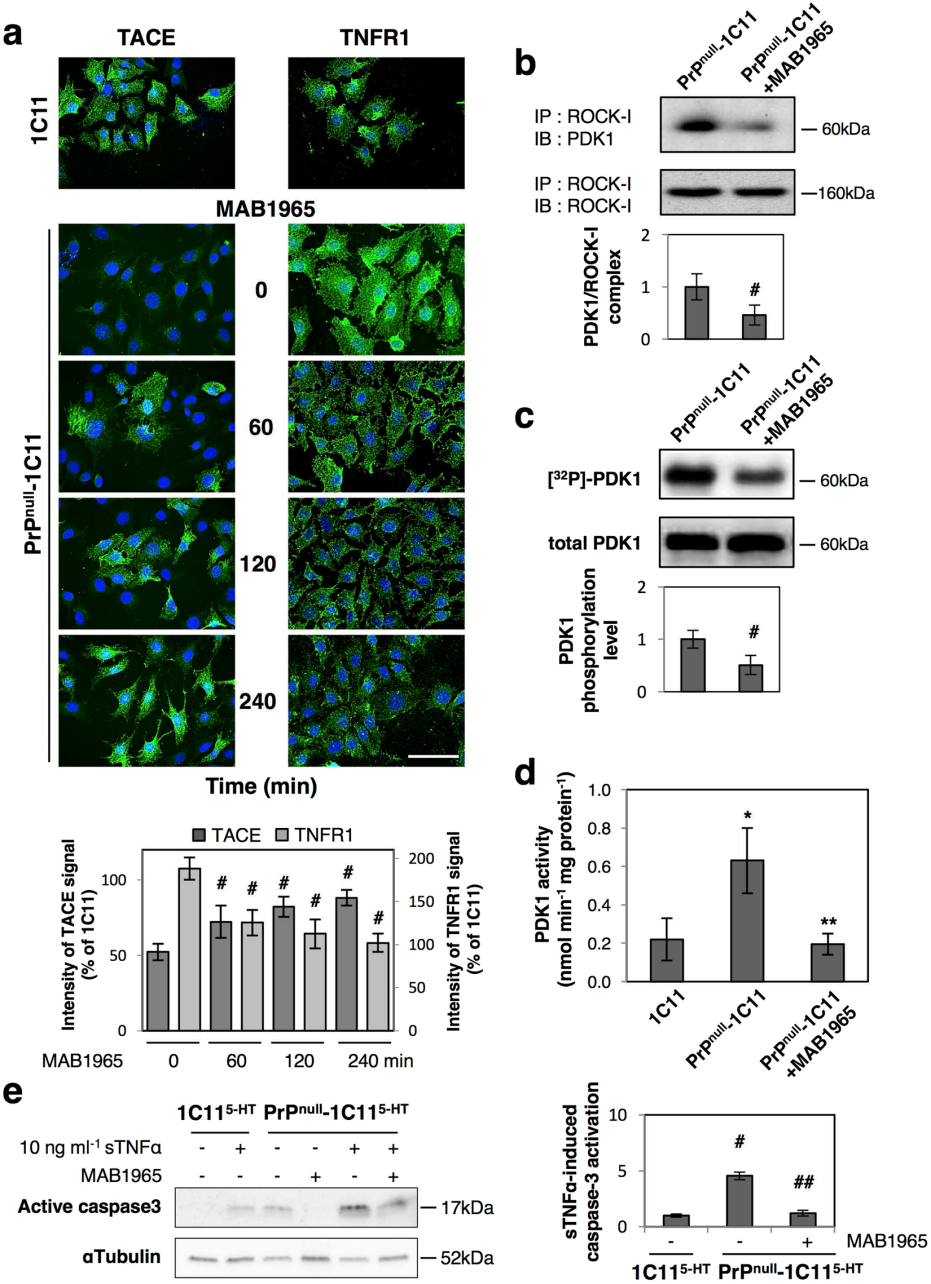
Misregulation of β1 integrin signaling in PrP^null^-cells causes TACE internalization and defect of TNFR1 shedding. (**a**) Immunolabeling experiments showing time courses of TACE relocation to the plasma membrane and concomitant rescue of TNFR1 shedding upon β1 integrin neutralization with MAB 1965 antibodies (1 µg ml^−1^). ^#^ *p* < 0.05 *vs*. nontreated PrP^null^-1C11 cells. (**b**) Immunoprecipitation of ROCK-I followed by PDK1 western-blotting indicating reduced ROCK-I and PDK1 interaction in PrP^null^-1C11 cells treated with neutralizing β1 integrin antibodies. ^#^ *p* < 0.05. (**c**) Neutralization of β1 integrins in PrP^null^-1C11 cells decreases phosphorylation of PDK1 as assessed by ^32^P metabolic labeling followed by PDK1 immunoprecipitation and western-blotting. ^#^ *p* < 0.05. (**d**) PDK1 activity returns to basal level in PrP^null^-1C11 cells exposed to MAB 1965 antibodies. *p < 0.01 *vs*. 1C11 cells. **p < 0.01 *vs*. untreated PrP^null^-1C11 cells. (**e**) Reduced sTNFα-induced caspase-3 activation in PrP^null^-1C11^5-HT^ cells exposed to MAB 1965 antibodies for 4 h. ^#^ *p* < 0.05 *vs*. PrP^C^ expressing 1C11^5-HT^-cells exposed to sTNFα. ^##^ *p* < 0.05 *vs*. PrP^null^-1C11^5-HT^ cells treated with sTNFα. Data shown are the mean ± SEM from three experiments performed in triplicate.

Exposure of PrP^null^-1C11 cells to neutralizing antibodies towards β1 integrins (MAB1965) relocated TACE to the plasma membrane of PrP^null^-1C11 cells (Fig. 4a), arguing that β1 integrin overactivity in the absence of PrP^C^ triggers TACE internalization. Redirection of TACE to the cell surface started after 60 min exposure to ΜΑΒ1965. Immunofluorescence experiments indicated that TACE signal measured at the plasma membrane of PrP^null^-1C11 cells after 120 and 240 min exposure to MAB1965 was comparable to that of PrP^C^ expressing 1C11 cells. Correlating TACE relocation to the cell surface, neutralization of β1 integrins rescued TNFR1 shedding as assessed by the progressive disappearance of TNFR1 immunostaining at the plasma membrane of PrP^null^-1C11 cells exposed to MAB 1965 (Fig. 4a). After β1 integrin neutralization for 120 to 240 min, cell surface TNFR1 level in PrP^null^-cells was highly comparable to that measured with PrP^C^ expressing cells. Manganese (Mn^2+^) is widely used to investigate conformational changes associated with the activation of integrins and the recruitment of signaling pathways^26, 27^, as Mn^2+^ binds integrins and strongly up-regulates integrin function by mimicking inside-out signaling events^28, 29^. In 1C11 and 1C11^5-HT^ cells expressing PrP^C^, forced stimulation of β1 integrin activity with 100 µM Mn^2+^ for 4 h promoted the internalization of TACE (Supplementary Fig. 3a,c) and abrogated TACE-mediated shedding of TNFR1 (Supplementary Fig. 3b,c) in a ROCK-I/PDK1-dependent manner. This set of experiments demonstrates that misregulation of β1 integrin signaling activity caused by loss of PrP^C^ regulatory action over β1 integrins (in PrP^null^-cells or in Mn^2+^-treated PrP^C^ expressing cells) is at the root of TACE internalization.

Finally, we monitored that restoration of TACE α-secretase at the plasma membrane and subsequent recovery of TNFR1 shedding in PrP^null^-cells exposed to the neutralizing β1 integrin antibody MAB1965 (240 min) were associated with disruption of the ROCK-I/PDK1 complex (Fig. 4b), reduced phosphorylation of PDK1 (Fig. 4c), decrease in PDK1 activity in PrP^null^-1C11 cells (Fig. 4d), and lower cell sensitivity to sTNFα, as inferred by the decreased sTNFα-induced activation of caspase-3 (Fig. 4e).

These overall data provide the prime evidence that loss of PrP^C^ regulatory function towards β1 integrin signaling and downstream overactivation of ROCK-I trigger (i) PDK1 overactivation, (ii) PDK1-dependent TACE internalization and (iii) abrogation of TACE shedding activity. By modulating β1 integrin signaling and activation of the ROCK-I/PDK1 module, PrP^C^ physiologically ensures bioavailability of active TACE α-secretase at the plasma membrane for TNFR1 shedding that thereby protects neuronal stem cells and neurons from sTNFα toxicity.

### Enhanced sensitivity of PrP^0/0^ mice to sTNFα inflammatory challenge can be counteracted upon PDK1 inhibition

To corroborate our *in vitro* data with the *in vivo* situation, we next probed in the brain of PrP^0/0^ mice the status of PDK1, the TACE shedding activity towards TNFR1, and the sensitivity to sTNFα-mediated inflammation.

**Figure 5:**
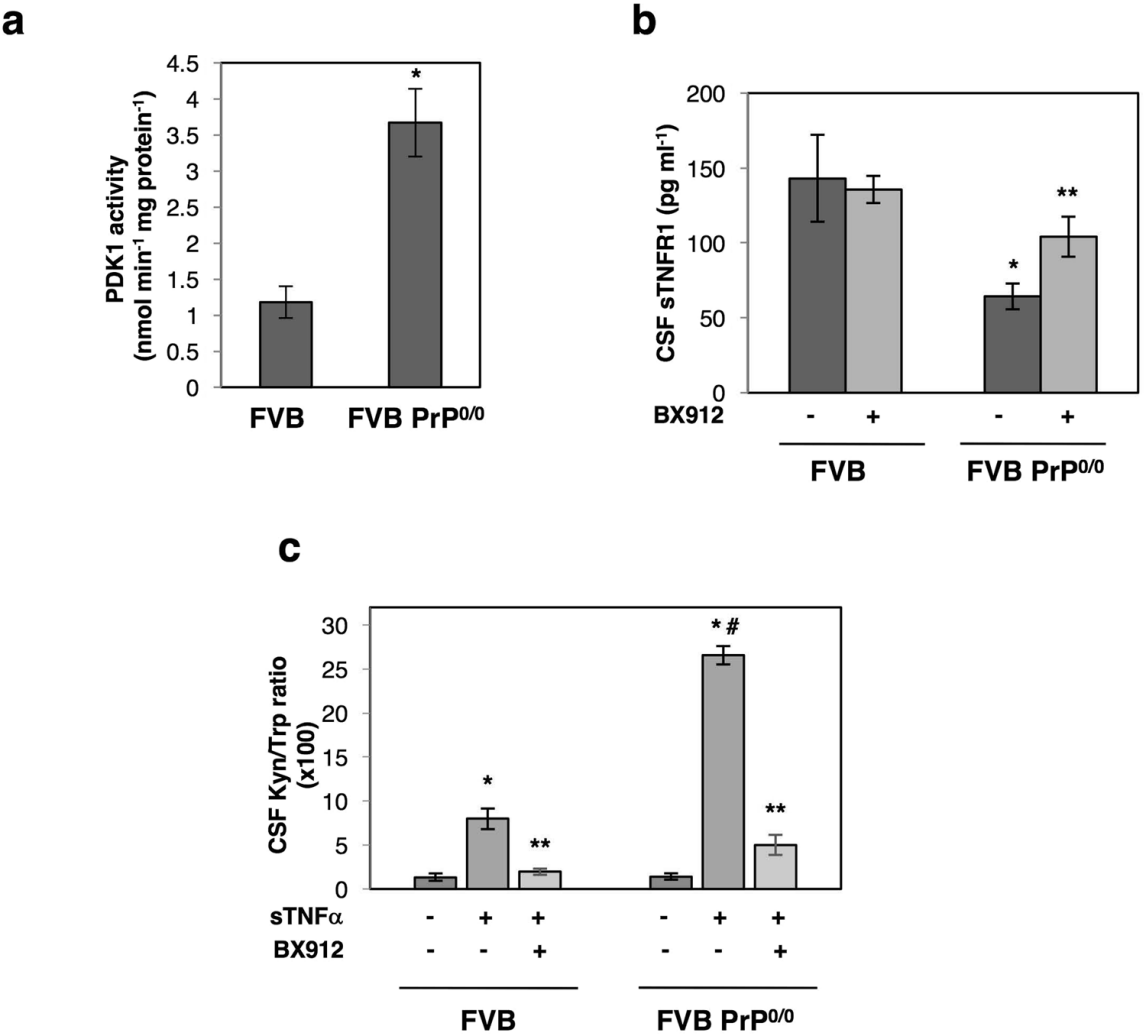
PDK1 inhibition protects FVB PrP^0/0^ mice from sTNFα-induced inflammation. (**a**) Measure of PDK1 activity indicating a rise of PDK1 activity in the brain of 20-weeks old FVB PrP^0/0^ mice compared to FVB wild type mice (n = 6 for each group). Values are means ± SEM. *p < 0.01 *vs*. FVB mice. **(b)** Concentration of sTNFR1 in the CSF of 20 weeks-old FVB PrP^0/0^ and wild type mice intracerebroventricular (icv) injected or not with the PDK1 inhibitor BX912 (n = 6 for each group). Values are means ± SEM. *p < 0.01 *vs*. nontreated FVB mice. **p < 0.01 *vs*. nontreated FVB PrP^0/0^ mice. **(c)** [kynurenine]/[tryptophan] ratio in the CSF of 20 weeks-old FVB PrP^0/0^ and wild type mice icv injected or not with BX912 following a sTNFα challenge (200 ng in 10 µl saline buffer) for 24 h (n = 6 for each group). Values are means ± SEM. *p < 0.01 *vs*. mice nontreated with sTNFα. ^#^ *p* < 0.01 *vs*. FVB mice treated with sTNFα. **p < 0.01 *vs*. sTNFα-treated mice.

First, we measured a ∼3-fold increase in PDK1 activity in brain extracts from 20 weeks-old FVB PrP^0/0^-mice (Fig. 5a) and a ∼2.5-fold decrease in soluble TNFR1 (sTNFR1) level in the cerebrospinal fluid (CSF) of FVB PrP^0/0^-mice (Fig. 5b) compared to their wild type counterparts. Intracerebroventricular (icv) injection of the PDK1 inhibitor BX912 (1 µM) in FVB PrP^0/0^-mice provoked a ∼2-fold increase in CSF sTNFR1 level (Fig. 5b), indicating that deficit of TNFR1 shedding in the brain of PrP^0/0^-mice originates from PDK1 overactivity.

We next challenged FVB PrP^0/0^-mice and their PrP^C^ expressing FVB counterparts (n = 6) with an icv dose of sTNFα (200 ng in 10 µl saline buffer) for 24 h. The neuroinflammation effect of sTNFα was evaluated through measure of the concentrations of kynurenine and tryptophan in the CSF as the kynurenine pathway of tryptophan metabolism was shown to mediate the action of pro-inflammatory cytokines, including sTNFα, in the brain^30–32^. Following the challenge with sTNFα, FVB animals expressing PrP^C^ showed a ∼7-fold increase in the CSF [kynurenine]/[tryptophan] ratio, while PrP^0/0^-animals showed an exaggerated response with a ∼17-fold elevation of the [kynurenine]/[tryptophan] ratio (Fig. 5c). This suggests that in the absence of PrP^C^ excessive TNFR1 signaling combined with deficit of the anti-inflammatory sTNFR1 factor caused by PDK1 overactivation would exacerbate the pro-inflammatory action of sTNFα in the brain. Accordingly, we showed that PDK1 inhibition and subsequent restoration of TNFR1 shedding (Fig. 5b) reversed the exaggerated kynurenine response induced by sTNFα icv injection in FVB PrP^0/0^ mice (Fig. 5c).

As a whole, these *in vivo* data indicate that loss of PrP^C^ is associated with a defect of TNFR1 shedding, which in turn exacerbates cell sensitivity to sTNFα-mediated inflammation.

## Discussion

Although corruption of normal function(s) of cellular prion protein (PrP^C^) plays a central role in TSEs, PrP^C^ role(s) remain(s) elusive. This study discloses that PrP^C^ limits the sensitivity of cells to the pro-inflammatory cytokine sTNFα by restricting the level of TNFR1 present at the plasma membrane. Such protective action of PrP^C^ towards TNFα toxicity depends on the signaling activity of PrP^C^ (i) to stimulate the cleavage of TNFR1 and the release of soluble TNFR1 (sTNFR1) through PrP^C^ coupling to the NADPH oxidase–TACE α-secretase pathway and (ii) to ensure active TACE bioavailability at the plasma membrane through negative control of β1 integrin coupling to ROCK-I and PDK1 kinases.

PrP^C^ acts as a signaling molecule at the cell surface and activates diverse effectors involved in neuronal homeostasis, including NADPH oxidase and TACE α-secretase^5, 8, 9^. Through coupling to the NADPH oxidase-TACE pathway, PrP^C^ promotes the release of sTNFα into the cell microenvironment^9, 33^. With 1C11-derived neuronal cells, sTNFα behaves as an autocrine modulator of neurotransmitter-associated functions devoid of any toxicity^9^. Here, we describe that PrP^C^ also takes part to the regulated cleavage of transmembrane TNFR1 and the subsequent release of sTNFR1 into the cell microenvironment through TACE activation. The identification of TNFR1 as a novel target of the PrP^C^/NADPH oxidase/TACE coupling sheds light on how PrP^C^ fine-tunes the cell response to sTNFα. By controlling the levels of shed TNFα and plasma membrane TNFR1, PrP^C^ thus confines the role of sTNFα to modulation of neuronal functions. Accordingly, due to the peculiar binding stoichiometry between sTNFR1 and sTNFα (2:3), sTNFR1 molecules released by PrP^C^ signaling (3000 molecules per 1C11^5-HT^ cell) neutralize PrP^C^-induced shed TNFα (4000 molecules per 1C11^5-HT^ cell) present in the cell microenvironment and, thereby, help to limit neuronal sTNFα signaling^34^. Dual control of TNFα release and TNFR1 shedding by TACE was also reported to protect liver from lipopolysaccharide (LPS)-induced inflammatory injury^35^. The present study also shows that the PrP^C^/TACE-mediated TNFR1 shedding ensures cell protection against an exogenous sTNFα insult with increased sensitivity of PrP^null^-cells and PrP^0/0^ mice towards sTNFα. This is in line with increased vulnerability of PrP^0/0^ mice to LPS-induced septic shock compared to PrP^C^ expressing mice^2^ associated with hyperactive inflammatory responses^36^. Our data add to the global idea that PrP^C^ exerts stress protection in a physiological context^37–40^ by adjusting the cell response to sTNFα of endogenous or exogenous origin.

We further evidence that the protective role of PrP^C^ towards sTNFα also depends on its capacity to maintain TACE α-secretase at the plasma membrane. The rise of sensitivity of PrP^null^-cells to exogenous sTNFα is associated with an increased level of TNFR1 molecules present at the plasma membrane caused by deficit of TACE activity. From a mechanistic point of view, defect of TACE shedding activity in PrP^null^-cells originates from the displacement of TACE from the plasma membrane to intracellular compartments. The internalization of TACE in the absence of PrP^C^ depends on a gain of plasma membrane β_1_ integrin signaling. Lowering β_1_ integrin activity in PrP^null^-cells directs TACE back to the plasma membrane and rescues TACE-mediated TNFR1 shedding. Acting as a scaffolding protein, PrP^C^ limits β1 integrin microclustering at the plasma membrane and negatively regulates β_1_ integrin signaling^6^. We further show that in the absence of PrP^C^ excessive β1 integrin signaling and downstream ROCK-I overactivity promote overactivation of PDK1, which in turn triggers TACE internalization. Our work thus supports the view that PrP^C^ cytoprotective effect against sTNFα toxicity is intimately linked to functional interactions between PrP^C^ and β1 integrin.

In prion diseases, it is now widely acknowledged that the subversion of PrP^C^ normal functions by PrP^Sc^ takes a critical part in neuronal cell demise^41–45^. Whether loss of PrP^C^ function upon its conversion into PrP^Sc^ or gain of PrP^C^ function by PrP^Sc^ lies at the root of neurodegeneration is still debated. The phenotypic proximity of PrP^null^-cells with prion-infected cells lends support for loss-of-PrP^C^ cytoprotective role towards TACE-mediated TNFR1 shedding along TSEs. Of note, is the increased sensitivity to sTNFα toxicity related to plasma membrane TNFR1 overexpression^13^ highly comparable between PrP^null^- and prion-infected cells. Correlatively, the overactivation of ROCK-I and PDK1, as well as subsequent internalization of TACE^13, 21^ occur with comparable intensities between PrP^C^-depleted and prion-infected cells. Such a loss-of-PrP^C^ cytoprotective function towards inflammation in TSEs is further supported *in vivo* by increase in PDK1 activity and deficit of TNFR1 shedding in the brain of PrP^0/0^ mice (Fig. 5a,b), as for prion-infected mice^13^. The exaggerated sensitivity to sTNFα-induced inflammation can be reversed upon PDK1 inhibition similarly between PrP^0/0^ mice (Fig. 5c) and prion-infected mice^13^.

Contrasting with other amyloid-based neurodegenerative diseases, inflammation in TSEs is atypical-qualified^46^ with low levels of inflammatory cytokines (sTNFα, IL1, IL6) released by activated microglia^47, 48^ in response to diverse signals emitted by prion-infected neurons^49^. Our data support the view that abrogation of PrP^C^ cytoprotective function against sTNFα by PrP^Sc^ is a priming event that renders prion-infected neurons sensitive to low doses of sTNFα. In line with this, the quickened death of prion-infected mice challenged with LPS^50, 51^ could be due to an accelerated degeneration of TNFR1 overexposing infected neurons provoked by the LPS-induced release of sTNFα by reactive microglial cells or peripheral production of sTNFα.

## Methods

### Antibodies

The mouse monoclonal SAF61 PrP antibody was from SPI-Bio (Montigny le Bretonneux, France). The rabbit polyclonal antibody to TNFR1 was from MBL International (Woburn, MA, USA). Rabbit polyclonal antibodies to TACE and active caspase-3 were purchased from QED Bioscience Inc. (San Diego, CA, USA) and Biovision (Mountain View, CA, USA), respectively. The rabbit polyclonal antibody to caveolin-1 (Cav-1) (610059) was obtained from Transduction Laboratories (Lexington, KY, USA). The rabbit polyclonal anti-MAP2 antibody and the mouse monoclonal neutralizing antibody towards β1 integrins (MAB1965) were from EMD Millipore (Darmstadt, Germany). The mouse monoclonal anti-actin antibody was from Novus Biologicals (Littleton, CO, USA). The rabbit monoclonal ROCK-I and polyclonal PDK1 antibodies were from Cell Signaling (Beverly, MA, USA). When non-specified, primary antibodies were used at 0.5 µg ml^−1^ for Western blot experiments and at 5 µg ml^−1^ for immunofluorescence experiments.

### Mice

Adult wild type FVB and PrP^0/0^ FVB mice were bred and underwent experiments, respecting European guidelines for the care and ethical use of laboratory animals (Directive 2010/63/EU of the European Parliament and of the Council of 22 September 2010 on the protection of animals used for scientific purposes). All animal procedures were approved by the Animal Care and Use Committee at Basel University (Switzerland).

### Treatment of mice and sample collection

Recombinant murine sTNFα (Biosource International, Camarillo, CA, USA) was intracerebroventricular (icv) injected at a dose of 200 ng in 10 µl saline buffer in combination or not with the PDK1 inhibitor (1 µM). At either 24 h following saline or sTNFα injection combined or not with BX912, mouse CSF was collected from the cisterna magna under anesthesia with 3% isoflurane.

### Measure of CSF tryptophan and kynurenine concentrations

CSF was analyzed by HPLC to quantify tryptophan (Trp) and kynurenine (Kyn). Briefly CSF samples were deproteinized by treatment with 86% methanol (1:6, vol/vol) to avoid Kyn diazotization induced by the usual acidic treatment of samples^52^. The resulting supernatant was filtered through 0.2 µm nylon membranes. Chomatography was performed with a ThermoFinnigan solvent delivery Spectra Series P100 pump. Sample injection was controlled by a Spark Holland Triathlon autosampler. A C18 reverse-phase HPLC column (Supelcosil LC-18-DB, 15 cm × 4.6 mm, 3 µm bead size; Supelco, Buchs, Switzerland) with a guard column (Supelguard LC-18-DB, 2 cm; Supelco, Buchs, Switzerland). Trp catabolites were eluted isocratically at a flow rate of 0.8 ml min^−1^ with a mobile phase consisting of a 94:6 mixture (by volume) of 16.2 mmol l^−1^ KH_2_PO_4_ and acetonitrile. The coulometric detection system consisted of a thin-layer flow-through electrochemical ESA Coulochem II detector connected to an ESA Model 5011 analytical cell containing two working electrodes made of porous graphite. The analytical cell voltage was set at +0.45 V for the first detector and +0.60 V for the second detector. Kyn and Trp were detected at +0.60 V. Chromatograms were generated and analyzed using D-7000 HPLC System Manager software.

### Soluble TNFα receptor type 1 (sTNFR1) quantification

The amount of soluble TNFR1 was measured in cell culture media or CSF by ELISA using the Mouse/Rat TNFR1/TNFRSF1A Quantikine ELISA kit (MRT10) according to the manufacturer’s instructions (R&D System, Minneapolis, MN, USA).

### Cell culture

1C11 cells were grown and induced to differentiate along the serotonergic (1C11^5-HT^) pathway as described in ref. 15. Primary CGNs were isolated from dissociated cerebella of 4–5 days-old FVB and PrP^0/0^ FVB mice as in ref. 53.

### Cell viability assays

The viability of ~1.10^5^ 1C11 or 1C11^5-HT^ cells expressing or not PrP^C^ exposed to recombinant murine sTNFα (Biosource International, Camarillo, CA, USA) was evaluated by the cellular reduction of 3-(4,5-dimethylthiazol-2-yl)-2,5-diphenyltetrazolium bromide (MTT, Invitrogen, Carlsbad, CA, USA)^13^.

Neuronal dysfunction in CGNs and PrP^0/0^-CGNs was evaluated by sTNFα-induced dendritic fragmentation. CGNs and PrP^0/0^-CGNs seeded (5.10^5^ cells per well) in 12-well plates coated with polyD-lysine (Sigma-Aldrich, St. Louis, MO, USA) were exposed to sTNFα. Cells were then fixed and stained with an anti-MAP2 antibody. After imaging with a fluorescence microscope (Zeiss Leica), cells exhibiting fragmented *vs*. non-fragmented dendrites were counted using ImageJ software (http://rsb.info.nih.gov/ij).

### Immunofluorescent experiments

Immunofluorescent labelings of PrP^C^, TNFR1, TACE and MAP2 were performed using standard protocols. Briefly, for cell surface detection of PrP^C^, TNFR1 and TACE, cells grown on glass coverslides were washed with cold PBS and fixed with 3.6% formaldehyde. Cells were incubated for 1 h at room temperature with the primary antibody in blocking buffer (PBS enriched with 2% FCS) and then with AlexaFluor 488-conjugated secondary immunoglobulins (1 µg ml^−1^; Molecular Probes, Eugene, OR, USA). For the intracellular detection of TACE or MAP2, cells fixed with 3.6% formaldehyde were permeabilized with 0.05% saponin or 0.1% Triton X-100, respectively, in PBS for 15 min at room temperature prior TACE or MAP2 immunostaining. Cell preparations were mounted under coverslips with Fluoromount G (Fisher Scientific, Pittsburgh, PA, USA) and analyzed by wide-field indirect immunofluorescence using a Leica DMI6000 B microscope (Wetzlar, Germany). For all images, out-of-focus haze was reduced by digital deconvolution of sets of 16 serial optical sections recorded at 0.3 µm intervals using the Adaptative Blind Deconvolution in the program Autoquant X (Meyer Instruments, Houston, TX, USA). All pixel values in each focal plane were then summed along z-axis to obtain the final image. Deconvoluted images were subjected to image analysis with the AQUA software^54^.

### Cell extract preparation and western blot analyses

Cells were washed in PBS/Ca/Mg and incubated for 30 min at 4 °C in lysis buffer (50 mM Tris-HCl pH 7.4, 150 mM NaCl, 5 mM EDTA, 1% Triton X-100, 1 mM Na_3_VO_4_ and protease inhibitors [Roche]). After centrifugation of the lysate (14,000 × g, 15 min), the concentration of the proteins in the supernatant was measured with the bicinchoninic acid method (Pierce, Rockford, IL, USA).

### PrP^C^ silencing and enzyme inhibition

We exploited 1C11 precursor cells stably expressing shRNA towards PrP^C^ in which PrP^C^ expression is repressed by more than 90% (referred to as PrP^null^-1C11 cells)^6^. Because PrP^null^-1C11 cells fail to implement a neuronal phenotype upon exposure to serotonergic inducers^6^, 1C11 cells were converted into serotonergic 1C11^5-HT^ neuronal cells and then transfected with a siRNA against PrP^C^ ^41^ using lipofectamine 2000 reagent following manufacturer’s instructions (Invitrogen, Carlsbad, CA, USA). These cells refer to as PrP^null^-1C11^5-HT^ cells.

NADPH oxidase activity was switched off using Diphenyleneiodonium (DPI). TACE activity was inhibited with TNFα processing inhibitor-2 (TAPI-2; Peptides International, Louisville, KY, USA). ADAM10 activity was blocked with GI254023X (Tocris Bioscience, Ellisville, MO, USA). γ-secretase activity was inhibited using DAPT (Tocris Bioscience, Ellisville, MO, USA). Guanylate cyclase activity was antagonized using NS-2028 (Tocris Bioscience, Ellisville, MO, USA). ROCK activity was inhibited with Y-27632 (Tocris Bioscience, Ellisville, MO, USA). PDK1 activity was switched-off with BX912 (Axon Medchem BV, Groningen, The Netherlands).

### Immunoelectron microscopy

Cells, grown to ~80% confluency, were rinsed twice with PBS, collected in PBS and 10 mM EDTA, and rinsed twice with PBS. The cell pellet was fixed with 0.2% phosphate-buffered glutaraldehyde for 20–120 s and blocked with bovine albumin. Processing of cells for ultrathin cryosectioning and immunolabeling was performed indirectly^55^, with 5- or 7-nm gold particles conjugated with affinity-purified goat anti-mouse or anti-rabbit IgG (Invitrogen, Carlsbad, CA, USA)^56^. The labeled specimens were negatively stained with sodium silicotungstate, and images were captured with a JEOL CX100 transmission electron microscope.

### RT-PCR analyses

RNA was isolated by using the RNase Easy Kit (Qiagen), including a RNase-free DNase I digestion step, as recommended by the manufacturer. For RT-PCR analysis, first-strand cDNA was synthesized from 5 µg of RNA with oligo(dT)17 primer, using 400 U of Superscript II reverse transcriptase (Invitrogen). PCR amplifications were then carried out in a 25 µl volume containing 1 µl of the reverse transcription products, using TaqDNA polymerase (Invitrogen). PCR products were analyzed on 1% agarose gels. Primers used for the PCR reactions included GAPDH, forward 5′-TGAAGGTCGGTGTGAACGGAT-3′ and reverse 5′-CATGTAGGCCATGAGGTCCAC-3′; TACE, forward 5′-CCAGCATCTGCTAAGTTGCTTCC-3′ and reverse 5′-CAGCACAGCTGCCAAGTCCTT-3′.

### ROCK-I immunoprecipitation

ROCK-I immunoprecipitation was performed according to standard protocols by using protein A-Sepharose beads (Amersham Pharmacia Biotech, Picataway, NJ, USA) coupled to anti-ROCK-I antibody and 100 μg of cell lysates or brain extracts. Immunoprecipitates were analyzed by western blotting using anti-ROCK-I and anti-PDK1 antibodies.

### Cell metabolic labeling with [^32^P]-orthophosphate

[^32^P]-orthophosphate labeling was performed as in ref. 57. Briefly, the cell culture medium was removed and cells were thoroughly washed with phosphate-free DMEM to eliminate any residual phosphate containing medium. [^32^P]-orthophosphate (40.7 Gbq mmol^−1^, GE Healthcare, Little Chalfont, UK) was added to the cell culture at a final concentration of 18.5 Mbq ml^−1^. After 2 h, the labeling medium was removed and the cells were lyzed after extensive washing.

### Measurement of PDK1 activity

PDK1 activity was measured in cell lysates or brain extracts using a fluorescent-labeled PDK1 substrate (5FAM-ARKRERTYSFGHHA-COOH, Caliper Life Sciences, Hanover, MD, USA)^58^. The relative amounts of substrate peptide and product phospho-peptide were determined using a Caliper EZ-reader (Caliper Life Sciences, Hanover, MD, USA).

### Data analysis

An analysis of variance of the cell/animal response group was performed using the Kaleidagraph software (Synergy Software, Reading, PA, USA). Values are given as means ± SEM. Significant responses (*P* < 0.05) are marked by symbols (#, *) and their corresponding p-values are provided in figure legends. When non-specified experiments were performed in three to five times in triplicates.

### Data availability

The datasets generated during and/or analysed during the current study are available from the corresponding author on reasonable request.

## Electronic supplementary material

Supplementary information
