# Supplementary material for: Protective role of cellular prion protein against TNFα-mediated inflammation through TACE α-secretase
Source: Sci Rep. 2017 Aug 9;7:7671. doi: 10.1038/s41598-017-08110-x (PMC5550509; doi:10.1038/s41598-017-08110-x)

SUPPLEMENTARY INFORMATIONS

Protective role of cellular prion protein against TNFα-mediated inflammation through TACE α-secretase

Juliette Ezpeleta, François Boudet-Devaud, Mathéa Pietri, Anne Baudry, Vincent Baudouin, Aurélie Alleaume-Butaux, Nathalie Dagoneau, Odile Kellermann, Jean-Marie Launay and Benoit Schneider

Supplementary Figure 1

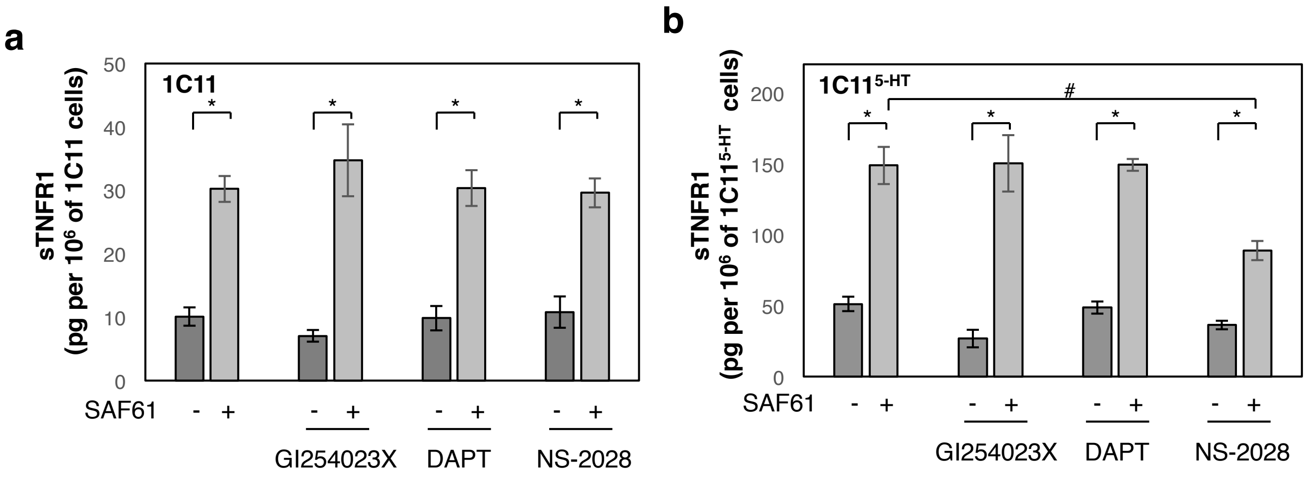

Supplementary Figure 1: Impact of the inhibition of guanylate cyclase, ADAM10 or γ-secretase on PrP^C-stimulated TNFR1 ectodomain shedding. ELISA-based measure of sTNFR1 concentration in the cell culture medium of 1C11 (**a**) and 1C11$^{5\text{-HT}}$ (**b**) cells treated with SAF61 PrP antibody (10 µg ml^{-1}) for 120 min in the presence or not of the guanylate cyclase inhibitor NS-2028 (1 µM), the ADAM10 inhibitor GI254023X (50 nM), or the γ-secretase inhibitor DAPT (10 µM). Data shown are the mean±SEM from three experiments performed in triplicate. **p*<0.01. #*p*<0.01.

Supplementary Figure 2

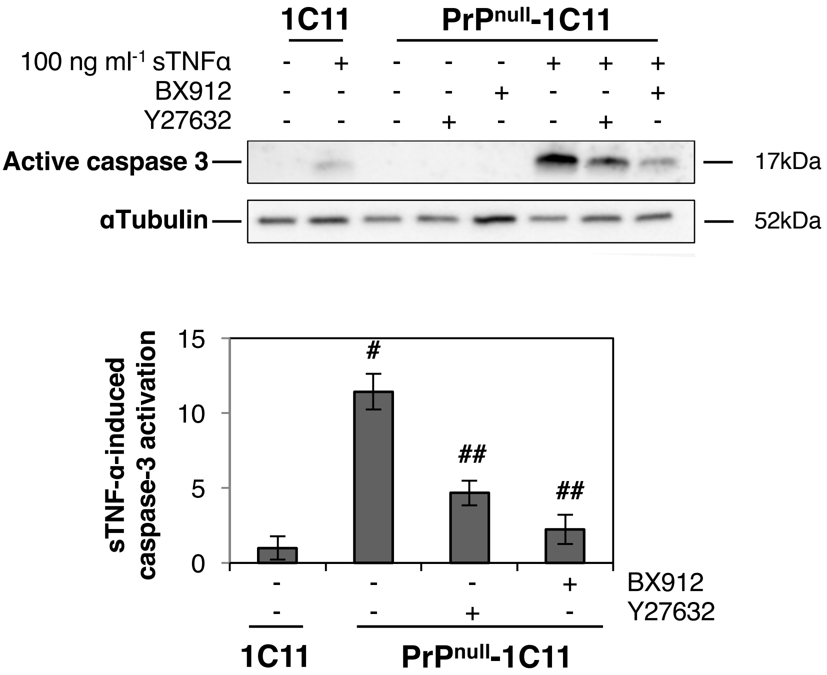

Supplementary Figure 2: Inhibition of ROCK-I or PDK1 protects PrP^{null}-1C11 cells from sTNFα toxicity. Western blots showing a stronger activation of caspase-3 in PrP^{null}-1C11 cells exposed to sTNFα (100 ng ml^{-1}) for 120 min than in 1C11 cells. Antagonizing either ROCK-I activity with Y-27632 (100 μM for 1h) or PDK1 activity with BX912 (1 μM for 1h) in PrP^{null}-1C11 cells reduces sTNFα toxic effect. Quantitative data are shown as the mean±SEM from three experiments performed in triplicate. #$p<0.05$ *vs.* 1C11 cells exposed to sTNFα; ##$p<0.05$ *vs.* PrP^{null}-1C11 cells treated with sTNFα.

Supplementary Figure 3

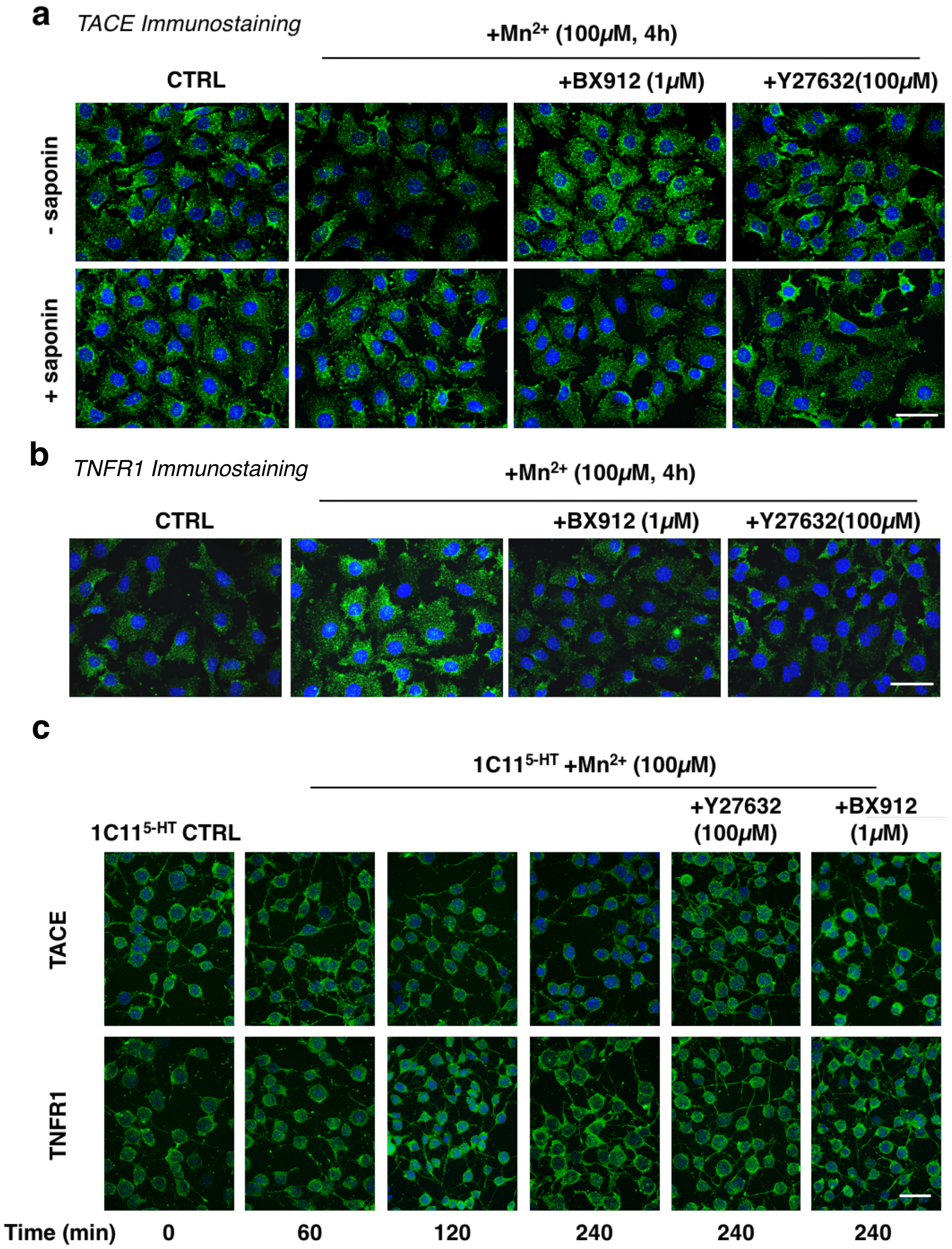

Supplementary Figure 3: Manganese-induced overstimulation of β1 integrin signaling triggers TACE internalization and TNFR1 shedding deficit in PrP^{C} expressing 1C11 and 1C11$^{5\text{-}HT}$ cells. Immunolabeling experiments showing internalization of TACE **(a)** and

increase in plasma membrane TNFR1 staining **(b)** in 1C11 cells exposed to Mn^{2+} (100 μM) for 4h that are counteracted upon inhibition of ROCK-I with Y-27632 (100 μM) or PDK1 with BX912 (1 μM). **(c)** Time-dependent reduction of TACE level and increase of TNFR1 amount at the surface of serotonergic $1C11^{5\text{-}HT}$ neuronal cells exposed to 100 μM Mn^{2+}. The inhibition of ROCK-I with Y-27632 (100 μM) or PDK1 with BX912 (1μM) blocks Mn^{2+} effects.

Supplementary Figure 4

Full-length gels from Figure 2e:

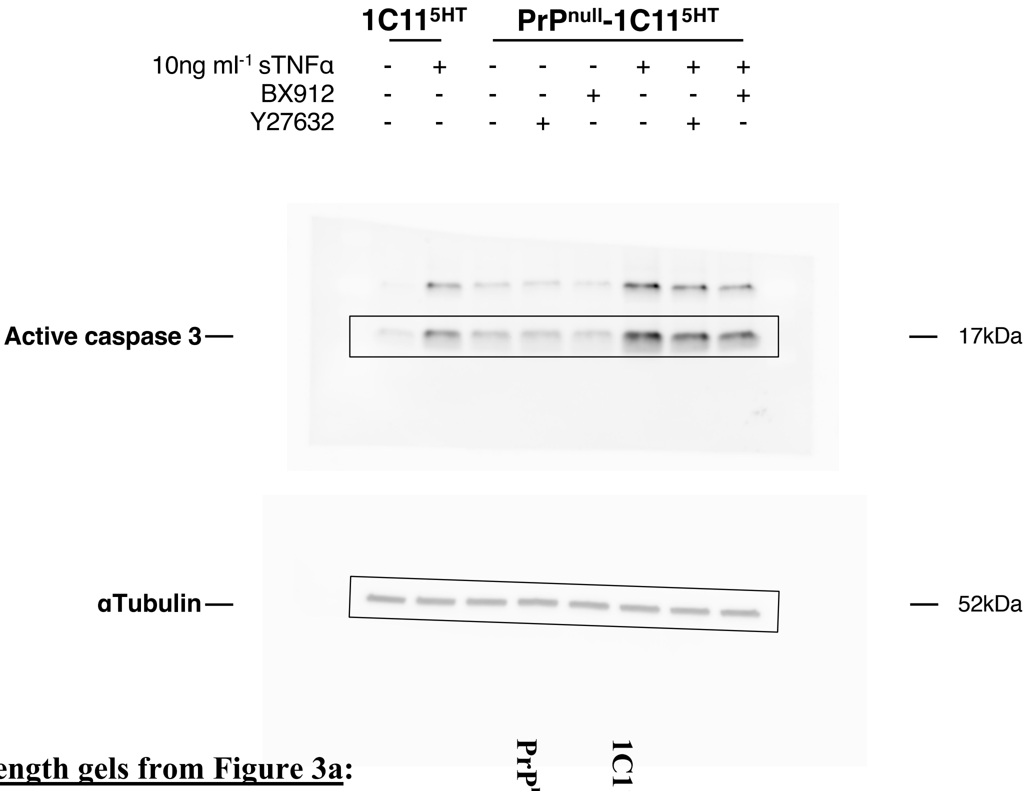

Full-length gels from Figure 3a:

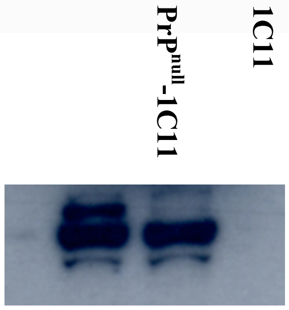

Full-length gels from Figure 3d:

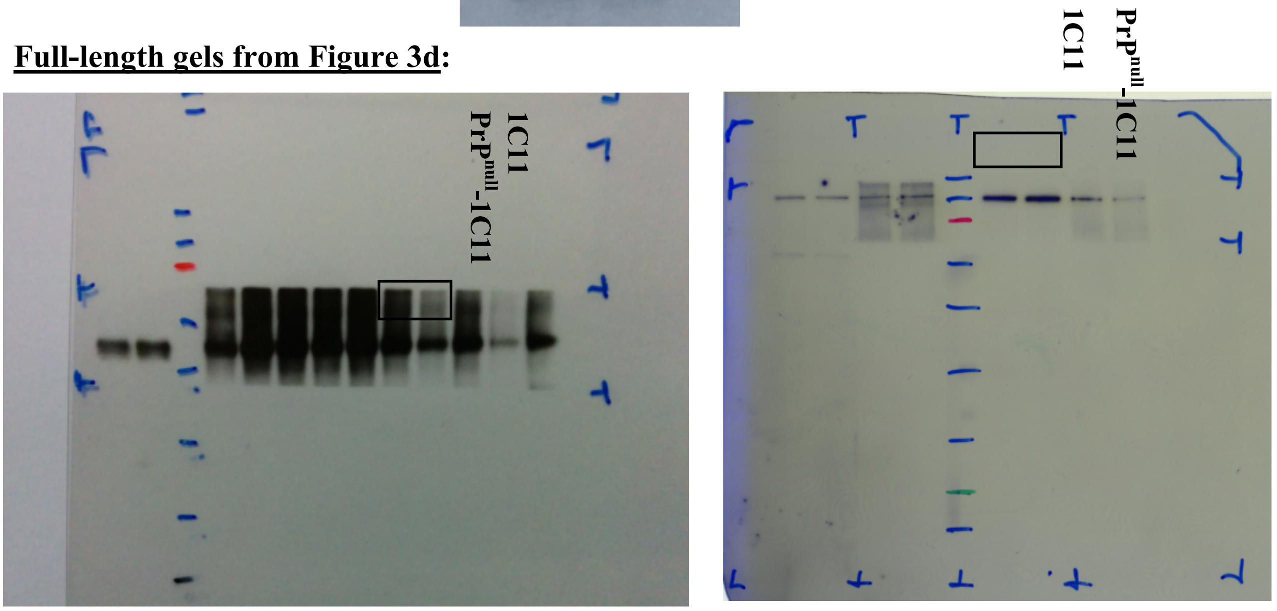

__**Full-length gels from Figure 4e:**__

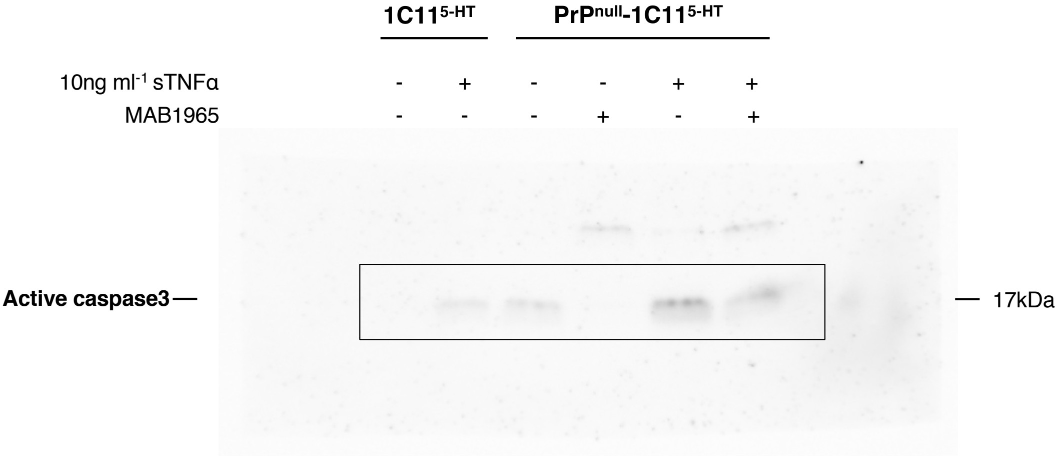

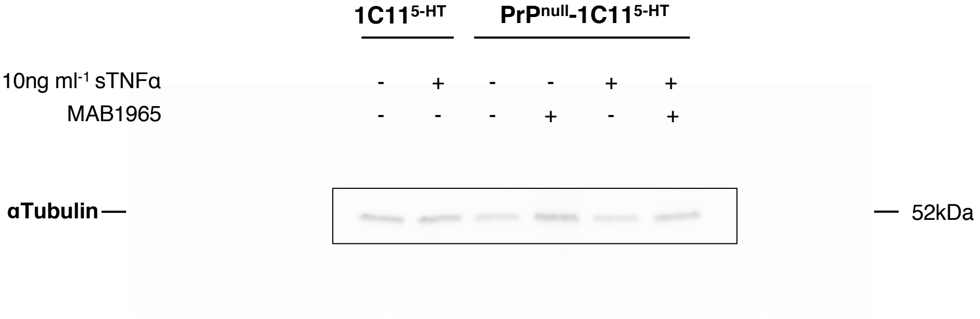

Supplement: Supplementary file 1 — Supplementary information [file 41598_2017_8110_MOESM1_ESM.pdf]
